# *Mycobacterium avium* Infection in a C3HeB/FeJ Mouse Model

**DOI:** 10.3389/fmicb.2019.00693

**Published:** 2019-04-03

**Authors:** Deepshikha Verma, Megan Stapleton, Jake Gadwa, Kridakorn Vongtongsalee, Alan R. Schenkel, Edward D. Chan, Diane Ordway

**Affiliations:** ^1^Mycobacteria Research Laboratories, Department of Microbiology, Immunology and Pathology, Colorado State University, Fort Collins, CO, United States; ^2^Department of Medicine, Denver Veterans Affairs Medical Center, Denver, CO, United States; ^3^Departments of Medicine and Academic Affairs, National Jewish Health, Denver, CO, United States; ^4^Division of Pulmonary Sciences and Critical Care Medicine, University of Colorado Anschutz Medical Campus, Aurora, CO, United States

**Keywords:** *Mycobacterium avium*, C3HeB/FeJ mouse model, immunity, pathology, nontuberculosis mycobacteria

## Abstract

Infections caused by *Mycobacterium avium* complex (MAC) species are increasing worldwide, resulting in a serious public health problem. Patients with MAC lung disease face an arduous journey of a prolonged multidrug regimen that is often poorly tolerated and associated with relatively poor outcome. Identification of new animal models that demonstrate a similar pulmonary pathology as humans infected with MAC has the potential to significantly advance our understanding of nontuberculosis mycobacteria (NTM) pathogenesis as well as provide a tractable model for screening candidate compounds for therapy. One new mouse model is the C3HeB/FeJ which is similar to MAC patients in that these mice can form foci of necrosis in granulomas. In this study, we evaluated the ability of C3HeB/FeJ mice exposure to an aerosol infection of a rough strain of MAC 2285 to produce a progressive infection resulting in small necrotic foci during granuloma formation. C3HeB/FeJ mice were infected with MAC and demonstrated a progressive lung infection resulting in an increase in bacterial burden peaking around day 40, developed micronecrosis in granulomas and was associated with increased influx of CD4^+^ Th1, Th17, and Treg lymphocytes into the lungs. However, during chronic infection around day 50, the bacterial burden plateaued and was associated with the reduced influx of CD4^+^ Th1, Th17 cells, and increased numbers of Treg lymphocytes and necrotic foci during granuloma formation. These results suggest the C3HeB/FeJ MAC infection mouse model will be an important model to evaluate immune pathogenesis and compound efficacy.

## Introduction

Infections due to nontuberculosis mycobacteria (NTM) are increasing worldwide, resulting in a **serious** public health problem (Prevots and Marras, 2015). NTM are mycobacterial species other than *Mycobacterium tuberculosis* complex and *Mycobacterium leprae*, that can cause pulmonary and extrapulmonary disease in vulnerable individuals, and are reported throughout the world (Prevots and Marras, 2015; Bryant et al., 2016). Given the ubiquitous nature of NTM in the environment, it is likely that repeated exposures from multiple sources- such as shower heads, swimming pools, and Jacuzzi baths and soil- increases the likelihood of established disease in susceptible individuals (Chan et al., 2010; Bryant et al., 2016). Other reports demonstrate the possibility that cystic fibrosis patients acquire NTM infection by contact to fomites or from patient to patient transmission (Bryant et al., 2016). Recent population-based studies have demonstrated this worldwide increase in NTM began in 2000 and currently in the United States, the prevalence of NTM lung disease exceeds that of tuberculosis by ∼10-fold (Epson et al., 2012; Prevots and Marras, 2015).

The most common NTM causing disease and outbreaks in the United States are species within the MAC—comprised of at least 9 species including *M. avium, M. intracellulare*, and *M. chimaera*, followed by *Mycobacterium abscessus* complex (including *M. abscessus* sensu stricto, *M. massiliense* and *M. bolletii*), *Mycobacterium chelonae* and *Mycobacterium kansasii* (De Groote and Huitt, 2006). The two most common preexisting conditions for NTM lung disease are emphysema and bronchiectasis, both of which may be acquired or heritable in origin (Chan et al., 2010; Prevots and Marras, 2015). Other host factors and phenotypes that are associated with NTM lung disease include advanced age, thin body habitus often with thoracic cage abnormalities such as scoliosis and pectus excavatum, gastroesophageal reflux, and use of inhaled corticosteroids and anti-tumor necrosis factor-alpha (anti-TNF-α) therapies (Shang et al., 2011; Bryant et al., 2016; Honda et al., 2018).

The two major clinical forms of MAC lung disease are manifested by two major radiographic patterns—the nodular bronchiectatic and fibrocavitary forms—which have differing lung pathology and bacterial burdens (Kartalija et al., 2013). MAC lung disease associated with upper lobe fibrocavitary pattern occurs dominantly in men with chronic obstructive pulmonary disease (COPD) (Glassroth, 2008). The fibrocavitary pattern is characterized by the formation of granulomas and increased bacterial burden (Gadkowski and Stout, 2008). The nodular-bronchiectasis form is often associated with immunocompetent women with granuloma formation in the airway walls and lower bacterial burdens (Glassroth, 2008). However, each type is not exclusively seen in one gender, and both types can be evident in a single patient (Okumura et al., 2008). The number of patients with MAC lung disease has increased worldwide, emphasizing the need for improved MAC modeling systems (Kartalija et al., 2013).

Treatment recommendations for MAC lung disease rely on a paucity of small clinical drug trials and mostly on expert opinion based on combined years of clinical experience (Griffith et al., 2007). Clarithromycin or azithromycin – used as part of a multidrug regimen – is currently the most important antibiotic in the treatment of MAC and many of the other NTM infections (Griffith et al., 2007; van Ingen et al., 2013). The present treatment guidelines for MAC lung disease recommend the combination of a macrolide (clarithromycin or azithromycin) with ethambutol and a rifamycin (rifampin), given for a minimum of 12 months after sputum culture conversion, with or without an aminoglycoside (streptomycin or amikacin given for the first several months) (Griffith et al., 2007; van Ingen et al., 2013). However, this recommended treatment regimen is only considered successful in roughly 50 to 60% of patients, with some recrudescence due to relapse and others due to a new infection from the environment (Griffith et al., 2007; van Ingen et al., 2013). The lack of success of the recommended treatment regimen (macrolide, ethambutol, rifamycin, aminoglycoside combination) is largely due to drug toxicities resulting in poor tolerability.

Four main animal models are routinely used for preclinical MAC experiments: C57BL/6, Balb/c, nude and beige mice infected by aerosol, intranasal, and intravenous exposure with MAC (Gangadharam et al., 1989; Pedrosa et al., 1994; Roger and Bermudez, 2001; Haug et al., 2013; Andrejak et al., 2015; Moraski et al., 2016; Blanchard et al., 2018). The majority of compound screening studies have been carried out with these mouse models due to their low cost and abundance of immunological reagents albeit a major drawback with them is a lack of necrotic granuloma formation (Abendano et al., 2014; Andrejak et al., 2015; Shin et al., 2015). In contrast, C3HeB/FeJ mice, develop highly organized encapsulated necrotic, hypoxic lesions following a *M. tuberculosis* infection (Kramnik et al., 2000; Obregon-Henao et al., 2013; Henao-Tamayo et al., 2015). Using a forward genetics approach, a region was identified as responsible for the 54.0-cM location of chromosome 1, termed the “super-susceptibility to tuberculosis-1” (*sst1*) locus (Kramnik et al., 2000). This locus was responsible for a reduced ability to control *M. tuberculosis* replication in the lungs. The susceptible *sst1* allele was also shown to be responsible for the formation of caseous necrosis in the lungs (Kramnik et al., 2000). The C3HeB/FeJ mouse model has been principally used to study compound screening, host immune response and vaccine efficacy with *M. tuberculosis* infection (Obregon-Henao et al., 2013; Henao-Tamayo et al., 2015) because they are capable of forming necrotic, hypoxic tubercle granulomas (Pichugin et al., 2009; Obregon-Henao et al., 2013; Henao-Tamayo et al., 2015). A MAC infection mouse model with the ability to develop necrotic granulomas is required to understand if a compound or vaccine-induced immune response can enter the site of necrosis to eradicate the bacilli.

Hence, we undertook a study to infect C3HeB/FeJ mice with an aerosol of a rough strain of *Mycobacterium avium* 2285 to determine if they develop a progressive infection and develop necrotic foci as well as characterize the cellular immune response. To better characterize the immune and pathologic responses induced by MAC infection in the C3HeB/FeJ mice would greatly improve the usefulness of this animal model for the testing of urgently needed new antimicrobial compounds and vaccines.

## Materials and Methods

### Mice

Specific pathogen-free female C3Heb/FeJ, 6 to 8 weeks old, were purchased from the Jackson Laboratories (Bar Harbor, ME). Mice were maintained in the biosafety level 3 facilities at Colorado State University and were given sterile water, chow, bedding, and enrichment for the duration of the experiments. The specific-pathogen-free nature of the mouse colonies was demonstrated by testing sentinel animals. All experimental protocols were approved by the Animal Care and Use Committee of Colorado State University.

### MAC Model

The *M. avium* 2285 strain with a rough colony morphology and positive for biofilm formation was obtained from a pulmonary MAC patient with a fibrocavitary form of disease (gift from Drs. Stephen Holland and Kenneth Olivier, National Institute of Allergy and Infectious Diseases). The *M. avium* 2285 strain was found to be of high virulence in Beige mouse model infected with a high dose aerosol (1.0 × 10^9^ CFU) (unpublished data). The inoculum was prepared by thawing the bacterial vial, sonicating for 10 to 15 s, and vortexing to remove any clumps that formed during freezing. Thereafter, the mycobacterial suspension was obtained from the vial with a 1-ml tuberculin syringe fitted with a 26.5-gauge needle and expelled back into the vial. This procedure was repeated into the vial 20 times without removing the needle to mix the suspension and break up any small clumps of bacilli (Ordway et al., 2008b; Obregon-Henao et al., 2015).

C3HeB/FeJ mice were infected with MAC using a Glas-Col aerosol generator (Glas-Col, Terre Haute, IN, United States), using the clinical isolate *M. avium* 2285 rough strain (henceforth referred to as MAC 2285) calibrated to deliver 2,000 bacteria into the lungs per mouse (Obregon-Henao et al., 2013; Henao-Tamayo et al., 2015). The following day, five mice were euthanized and their whole lungs, spleens, and livers were harvested to determine the baseline bacterial burden. The organs were homogenized in phosphate-buffered saline (PBS), and serial dilutions were plated on nutrient 7H11 agar and tryptic soy agar (TSA) for 3 weeks at 37°C, then CFU were enumerated (Bryant et al., 2016).

The dose of MAC 2285 resulted in progressive infection without showing signs of mortality at the 2,000 CFU. The increased susceptibility of the C3HeB/FeJ mice to mycobacteria justified the use of lower infective doses of MAC 2285 compared to the other more resistant mice (C57BL/6, Balb/c, nude or beige) which require an infective dose of 1 × 10^8^–1 × 10^11^ CFUs (Ji et al., 1994; Andrejak et al., 2015).

### Animal Infection

Using a Glas-Col aerosol generator, C3HeB/FeJ mice were infected by an aerosol of the clinical isolate MAC 2285 rough strain calibrated to deliver 2,000 bacteria into the lung, spleen and liver per mouse (Obregon-Henao et al., 2013; Henao-Tamayo et al., 2015). The whole lung, spleen, and liver from the mice for each condition at each time point (n = 5) were harvested to quantify bacterial burden (lung, spleen and liver, n = 5), histological analysis (lungs, n = 5), and flow cytometric analyses (lung and spleen, n = 5) at 20, 30, 40, 50, and 60 days post-challenge. In brief, bacterial loads were determined by plating whole organ serial dilutions of organ homogenates onto nutrient 7H11 and TSA agar plates. The rationale for using both 7H11 and TSA plates are any additional environmental NTM contamination of our CFUs can be identified on the TSA plates. Colony forming units (CFU) were quantified after incubation for 3 weeks at 37°C. The tissues of additional groups of mice were analyzed for pathological and immune response analysis. The results shown in this study are representative of two independent experiments using five animals per time point.

### Histological Analysis

The whole lung lobe from each mouse was fixed with 10% formalin in PBS. Sections from these tissues were stained with haematoxylin-eosin and with Ziehl-Neelsen acid-fast stains (Obregon-Henao et al., 2013; Henao-Tamayo et al., 2015).

### Lung and Spleen Cell Digestion

Briefly, single cell suspensions were prepared as described previously (Ordway et al., 2008a; Shang et al., 2011; Obregon-Henao et al., 2013). The lungs and spleens were aseptically removed, teased apart and treated with a solution of deoxyribonuclease IV (DNAse) (Sigma Chemical, 30 μg/ml) and collagenase XI (Sigma Chemical, 0.7 mg/ml) for 45 min at 37°C. To obtain a single-cell suspension, the organs were gently passed through cell strainers (Becton Dickinson, Lincoln Park, NJ, United States). Any remaining erythrocytes were lysed with Gey’s solution (0.15 M NH_4_Cl, 10 mM KHCO_3_) and the cells were washed with Dulbecco’s modified Eagle’s minimal essential medium. Cell suspensions from each individual mouse were incubated with monoclonal antibodies to various cytokines and cell surface markers labeled with fluorescein isothiocyanate (FITC), phycoerythrin (PE), peridinin chlorophyll-a protein (PerCP), or allophycocyanin (APC) at 4°C for 30 min in the dark as described previously (Ordway et al., 2008a; Shang et al., 2011; Obregon-Henao et al., 2013). Total cell numbers were determined by flow cytometry using BD^TM^ Liquid Counting Beads, as described by the manufacturer (BD PharMingen, San Jose, CA, United States). All analyses were performed with an acquisition of at least 100,000 total events for T cells and 200,000 total events for antigen-presenting cells.

### Cell Surface and Intracytoplasmic Cytokine Staining

Cells were first stained for cell surface markers as indicated above and thereafter the same cell suspensions were prepared for intracellular staining as described previously (Ordway et al., 2007, 2008a; Obregon-Henao et al., 2013). For flow cytometry analysis, single-cell suspensions prepared from the lungs of naïve and MAC 2285 infected mice were re-suspended in PBS containing 0.1% of sodium azide. Cells were incubated in the dark for 25 min at 37°C with pre-determined optimal titrations of specific antibody (directly conjugated to FITC, PE, PerCP, APC, Pacific Blue, or Alexa 700); or after biotin antibody incubations washed and incubated for 25 min more with streptavidin Qdot800 (Invitrogen), followed by two washes in PBS containing 4% sodium azide. Measurement of intracellular cytokines was conducted by pre-incubating lung cells with monensin (3 μM) (Golgi Stop, BD PharMingen), anti-CD3 and anti-CD28 (both at 0.2 μg/10^6^ cells) for 4 h at 37°C, 5% CO_2_. The cells were then surface stained, incubated for 30 min at 37°C, washed, fixed and permeabilized with Perm Fix/Perm Wash (BD Pharmingen). Finally, the cells were stained with fluorescently-labeled antibodies directed against intracellular Foxp3 (FJK-16s), IL-17 (clone N49-653) and IFN- γ many of the IFN-γ (clone B27) and separately with their respective isotype controls (BD Pharmingen) for a further 30 min. All the samples were run on a Becton Dickinson LSR-II and data were analyzed using FACSDiva v5.0.1 software. Cells were gated on lymphocytes based on characteristic forward and side scatter profiles. Individual cell populations were identified according to their presence of specific fluorescent-labeled antibodies. All the analyses were performed with a minimum acquisition of 100,000 events for T cells and 200,000 events for macrophages and dendritic cells.

### Statistical Analysis

Data are presented using the mean values from 5 mice per group performed in duplicate experiments. The Student *t*-test was used to assess statistical significance between groups of mice. In addition, bacterial burdens for each experimental condition were analyzed with GraphPad Prism version 4 (GraphPad Software, San Diego, CA), using analysis of variance (ANOVA) comparison test. Data are presented using the mean values (*n* = 5) plus or minus the standard error of the mean (SEM). Significance was considered with a *P* value of < 0.05 (Obregon-Henao et al., 2015).

## Results

### Course of MAC Infection in C3HeB/FeJ Mice

Our goal was to assess the course of an aerosol infection during acute and chronic infection with MAC 2285 in mice. We evaluated aerosol exposure of a clinical, drug- resistant strain of MAC 2285 (∼2,000 bacilli per mouse) to determine if there is a progressive infection in the C3HeB/FeJ during the acute and relative chronic phases after infection.

**FIGURE 1:**
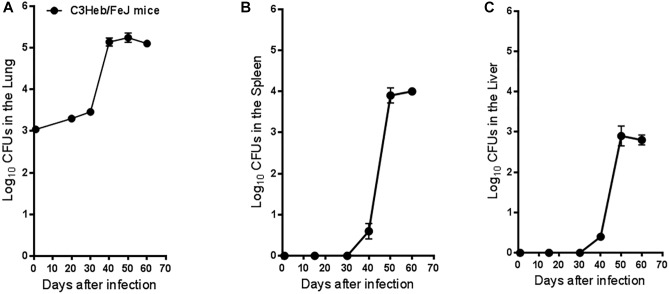
*Mycobacterium avium* infection in C3HeB/FeJ mice. Bacterial counts in the lungs **(A)**, spleens **(B)**, and livers **(C)** of C3HeB/FeJ mice infected with 2,000 MAC 2285 rough strain per mouse are shown. CFU were determined at 1, 20, 30, 40, 50, and 60 days after infection by plating serial dilutions of organ homogenates on nutrient 7H11 and TSA agar and quantifying CFU after 3 weeks incubation at 37°C. The C3HeB/FeJ mice after 40 days of infection showed increased bacterial burden ∼4.5 to 5.0 log_10_ in the lungs followed by bacterial burden plateauing during chronic phase of infection ∼50–60 days **(A)**. The C3HeB/FeJ mice showed a delay in bacterial dissemination in both the spleens **(B)** and liver **(C)** peaking after ∼50–60 days to ∼4.0 log_10_ CFU in the spleen and ∼3.0 log_10_ CFU in the liver. Results represent the average of two experiments (*n* = 5 mice per time point) and are expressed as log_10_ CFU (±SEM).

Mice infected with MAC were evaluated for bacterial loads in the lungs (Figure 1A), spleens (Figure 1B), and livers (Figure 1C) after 1, 20, 30, 40, 50, and 60 days of infection.

C3HeB/FeJ mice showed an increase in bacterial burden 40 days after infection, peaking at ∼5.2 log_10_ CFU in the lungs (Figure 1A), followed by bacilli persisting in the lungs and plateauing at ∼5.1 log_10_ CFU during the chronic phase of disease. C3HeB/FeJ mice showed a delayed dissemination to the spleens (Figure 1B), and livers (Figure 1C) over the first 30 days of infection, followed by an increase of ∼4.0 log_10_ CFU in the spleens peaking at 50 days and ∼3.0. log_10_ CFU in the livers during the chronic phase of disease.

These results demonstrate that aerosol exposure of MAC can progress slowly over the acute and subacute phases of disease, reaching a peak in bacterial burden in the lungs ay day 40. Furthermore, by the chronic phase of the infection, the bacterial burden remained persistently elevated in all the organs examined (Kitahara et al., 1997; Boyle et al., 2015).

### Development of Pathology in MAC Infection in C3HeB/FeJ Mice

**FIGURE 2:**
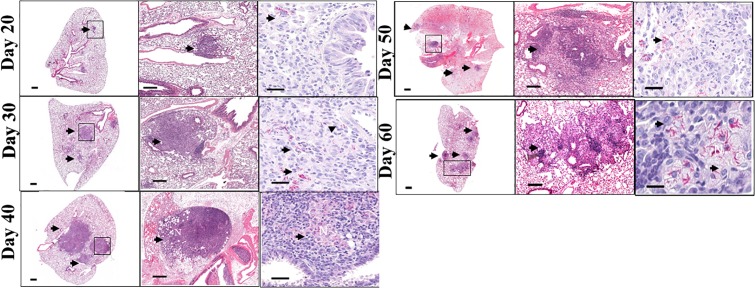
Pulmonary pathology in *Mycobacterium avium* infection C3HeB/FeJ mice. Lung pathology of MAC-infected C3HeB/FeJ mice. Shown are representative photomicrographs of haematoxylin-eosin-stained (left and middle columns) and of acid-fast stain (right column) of the lungs of MAC -infected C3HeB/FeJ mice. As early as 20–30 days after infection, small granulomas are evident in C3HeB/FeJ mice. As disease progressed (day 40), significant increase in granuloma size and bacterial burden (denoted by acid-fast staining) are found. During chronic phase of infection between 50 and 60 days the number of granulomas increased with increased clusters of acid-fast-staining bacilli, accumulating in areas of necrosis (N) (arrows). Magnifications, 1X (left), 20X (middle) and 100X (right).

The lung histopathology and acid-fast staining over the course of infection are shown in Figure 2 (left / middle and right columns, respectively). As early as day 40 after infection, C3HeB/FeJ mice developed small pulmonary necrotic lesions (Figure 2, left / middle columns), and multiple extracellular clusters of bacilli were observed within these lesions (Figure 2, arrows). During the chronic stage, the lungs of MAC -infected C3HeB/FeJ mice developed increased numbers of granulomas, greater numbers of intracellular and extracellular acid fast bacilli and progressive development of lesions, with notable lung tissue inflammation and small areas of necrosis (Figure 2 day 40–60). During the later stages of chronic disease much of the lung tissue was grossly consolidated, with inflammation and necrosis was evident. Overall, MAC infection of C3HeB/FeJ mice demonstrate disease progression resulting in pulmonary inflammation, lung consolidation and small foci of necrosis in pulmonary lesions.

### Kinetic Influx of the T Cell Response in *M. avium* Infected C3HeB/FeJ Mice

**FIGURE 3:**
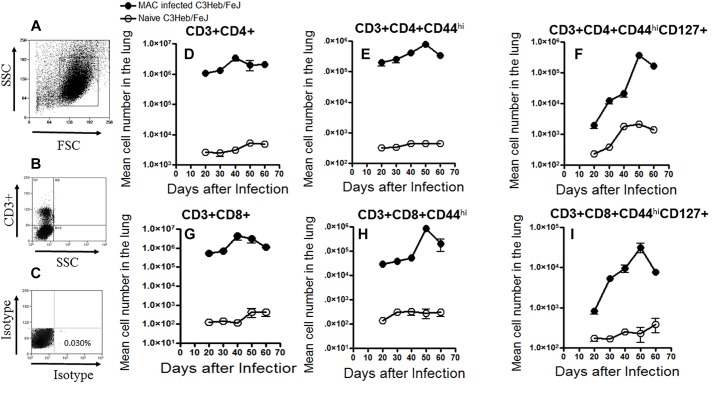
Kinetic influx of CD4 and CD8 T cell effector and memory cells in *M. avium*-infected C3HeB/FeJ mice. Increased percentages of activated effector and memory T cells were present after a moderate-dose infection of MAC 2285 rough in C3HeB/FeJ mice analyzed by flow cytometry compared to naïve controls. T cells were gated with a primary gate on viable FSC^low^ vs. SSC^low^ lymphocytes compared to the isotype controls **(A–C)** and then on CD3^+^ T cells, and analyzed for changes in the total mean cell number of CD3^+^CD4^+^ and CD3^+^CD8^+^ **(D,G)** cells over the course of infection. Shown are the numbers of activated effector T cells (CD4^+^CD44^hi^ and CD8^+^CD44^hi^) **(E,F)** and memory T cells (CD4^+^CD44^hi^CD127^+^ and CD8^+^CD44^hi^CD127^+^) **(F,I)** migrating to the lungs of C3HeB/FeJ mice peaking after 40–50 days of infection. During the later stages on day 60 of infection, C3HeB/FeJ mice expressed diminished numbers of activated effector and memory T cells. Results represent the mean number of cells of five mice for each condition from two independent experiments (±SEM).

T cells were gated with a primary gate on viable FSC^low^ vs. SSC^low^ lymphocytes and then on CD3^+^ T cells and compared to the isotype controls (Figure 3A–C), and analyzed for changes in the total mean cell number of CD3^+^CD4^+^ and CD3^+^CD8^+^ cells over the course of infection.

T cells that migrated to the lungs of naive control and MAC-infected C3HeB/FeJ mice were harvested and analyzed by flow cytometry. Specifically, naive CD3^+^CD4^+^ and CD3^+^CD8^+^ T cells, as well as the kinetic influx of activated T effector cells (CD3^+^CD4^+^CD44^hi^ and CD3^+^CD8^+^CD44^hi^) and effector memory T cells (CD3^+^CD4^+^CD44^hi^CD127^+^ and CD3^+^CD8^+^CD44^hi^CD127^+^) were determined in naive mice and MAC-infected C3HeB/FeJ mice (Henao-Tamayo et al., 2014). As expected, at days 30 and 40, when bacterial burden increased (Figure 1A) and pathology began to develop (Figure 2), increased numbers of (CD3^+^CD4^+^ and CD3^+^CD8^+^ T cells) were already found in MAC-infected C3HeB/FeJ mice compared to naive mice (Figure 3D,G). In addition, in MAC infected C3HeB/FeJ mice the (CD3^+^CD4^+^CD44^hi^ and CD3^+^CD8^+^CD44^hi^) activated T effector cells showed a delay in trafficking into the lungs peaking on day 50 compared to the overall CD4^+^ and CD8^+^ populations (Figure 3E,H). Similarly, in the MAC infected C3HeB/FeJ mice, the effector memory T cells (CD3^+^CD4^+^CD44^hi^CD127^+^ and CD3^+^CD8^+^CD44^hi^CD127^+^) cells demonstrated a lag in reaching the lungs, peaking on day 50 compared to the overall CD4^+^ and CD8^+^ populations, thereafter, these memory T cells declined (Figure 3F,I).

### Kinetic Influx of CD4 and CD8 T Cells Expressing IFN-γ, IL-17, and Foxp3 in MAC-Infected C3HeB/FeJ Mice

**FIGURE 4:**
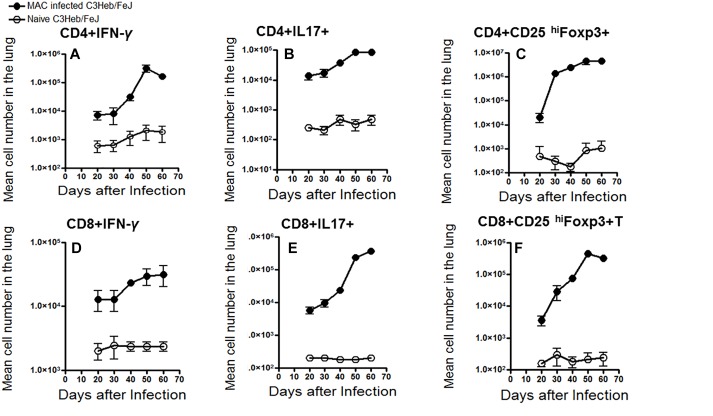
Kinetic influx of CD4 and CD8 T cells expressing IFN-γ, IL-17, and Foxp3 in *M. avium*-infected C3HeB/FeJ mice. Lung cells obtained from MAC-infected C3HeB/FeJ and naive control mice were analyzed by flow cytometry. **(A–C)** show CD4^+^ effector cells expressing IFN-γ, IL-17 and CD25^hi^Foxp3. MAC-infected C3HeB/FeJ mice showed increased numbers of CD4^+^IFN-γ^+^ and CD4^+^IL-17^+^ producing cells peaking between 40 and 50 days after infection with concomitant increased numbers of CD4^+^CD25^hi^Foxp3^+^ cell compared to the naïve mice. **(D–F)** show CD8^+^ effector cells expressing IFN-γ, IL-17, and CD25^hi^Foxp3. Interestingly, CD8^+^IFN-γ^+^ effector cells demonstrated decreased migration to the lungs with a concomitant increased number of CD8^+^IL-17^+^ and CD8^+^CD25^hi^Foxp3^+^ cells compared to the naïve mice. The data are expressed as the mean number of pulmonary cells in each organ ± SEM (*n* = 5 mice for each condition from two independent experiments (±SEM).

The kinetic influx of IFN- γ -producing CD3^+^CD4^+^ and CD3^+^CD8^+^ T cells, IL-17 producing CD3^+^CD4^+^ and CD3^+^CD8^+^ T cells, and suppressive T regulatory cells (CD3^+^CD4^+^ and CD3^+^CD8^+^ CD25^+^Foxp3^+^), were determined in the lungs of the MAC infected C3HeB/FeJ mice compared to naive mice. Interestingly, after day 40–50, when bacterial burden (Figure 1A) and pathology (Figure 2) were increasing, higher numbers of CD4^+^ IFN-γ-producing T cells were found in the lungs which declined at 60 days (Figure 4A,D). However, with respect to CD8^+^ IFN-γ-producing T cells in C3HeB/FeJ mice, what was observed to be overall lower were observed and increased slightly between 40 and 50 days over the course of the infection compared to naive mice (Figure 4B). MAC-infected C3HeB/FeJ mice demonstrated increased numbers of CD4^+^ and CD8^+^ T cells producing IL-17 (Figure 4B,E), and regulatory T cell markers (CD25^hi^ and Foxp3) (Figure 4C,F), that were associated with increased bacterial burden and organ pathology.

### Kinetics of Macrophages and Dendritic Cells in *M. avium*-Infected C3HeB/FeJ Mice

**FIGURE 5:**
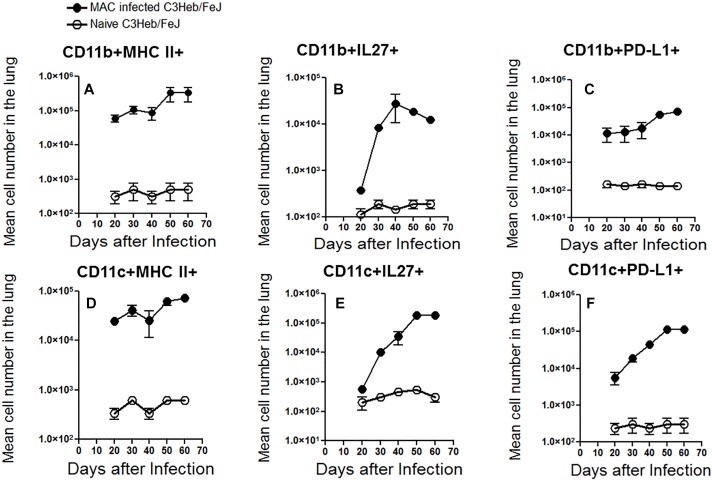
Kinetic influx of CD11b^+^ macrophages and CD11c^+^ dendritic cells in *M. avium-*infected C3HeB/FeJ mice. After the indicated times of MAC infection of C3HeB/FeJ mice and naïve mice, the lung cells obtained were analyzed by flow cytometry. Kinetic influx of CD11b^+^ macrophages and CD11c^+^ dendritic cells expressing MHC-II^+^ **(A,D)**, IL-27^+^ **(B,E)** and PD-L1^+^ **(C,F)** of MAC infected C3HeB/FeJ mice at the indicated times. The data are expressed as the mean number of pulmonary cells in each organ ± SEM (*n* = 5 mice per group). Results represent the mean number of cells of five mice for each condition from two independent experiments (±SEM).

Flow cytometric analysis was performed on MAC- infected C3HeB/FeJ mice and naïve mice in order to analyze the influx of CD11b^+^ macrophages and CD11c^+^ dendritic cells into the lungs. Between 40 and 50 days, a time at which the infection progressed, we observed a relative increase in cells staining positive for CD11b^+^ and CD11c^+^ MHC class II expression compared to the naïve mice (Figure 5A,D). This was consistent with the presence of increasing numbers of CD11b^+^ and CD11c^+^ cells expressing IL-27 (Figure 5B,E) and programmed death-ligand 1 (PD-L1) markers although both plateaued at 50 days of infection (Figure 5C,F) related with T cell immune suppression (Shu et al., 2017).

The slow increase of MHC II^+^ macrophages and dendritic cells between 40 and 50 days of infection, was associated with an increase number of CD4^+^ T cells producing IFN-γ and IL-17 increased arriving in the lungs at this later time range, whereas there was only a modest increase in the number of CD8^+^ T cells that produced IFN-γ (Figure 4D).

## Discussion

While MAC is less virulent than *M. tuberculosis*, these NTM are capable of causing chronic lung disease. Thus, development of a sustained progressive MAC infection in a mouse strain is an important endeavor to not only better characterize the immunopathogenesis in an *in vivo* model, but to also test candidate antimicrobials. Previous work using immunocompetent mouse strains demonstrated rapid clearance with lower amounts of MAC (Gangadharam, 1995), making such models suboptimal for more chronic human lung infection. In a MAC aerosol model using high doses of bacteria (1 × 10^9^–1 × 10^11^ CFU per mouse) in comparing BALB/c, C57BL/6, Nude and Beige mice, the Nude mice were identified as the most sensitive mouse strain to MAC while treatment efficacy was most noticeable in BALB/c mice (Torrelles et al., 2002; van Ingen et al., 2013; Andrejak et al., 2015; Cha et al., 2015). Beige mice are not uncommonly utilized as an *in vivo* mouse model for MAC infection and display many immune deficiencies similar to those occuring in AIDS patients such as a dominant Th2 response resulting in enhanced susceptibility to infection with NTM following either intravenous or aerosol infection (Caverly et al., 2015). Indeed, dissemination of MAC from gut of the Beige mice was shown to be more rapid than that seen in wildtype C57BL/6 mice (Torrelles et al., 2002). The Beige mice also had a defect in the influx of neutrophils to the site of infection and transfusing exogenous neutrophils mitigated their susceptibility; neutrophil depletion studies with wildtype C56BL/6 mice demonstrated increased susceptibility (Torrelles et al., 2002). Subsequent studies in another mouse strain revealed that a defect in CXCR2 chemokine signaling impaired the early and rapid recruitment of neutrophils in response to MAC infection (Torrelles et al., 2002; van Ingen et al., 2013). Nevertheless, the initial discovery and further confirmation of sustained MAC disease in the Beige mouse have encouraged many investigators to use this model to screen potential chemotherapeutic compounds for the treatment of MAC disease (Obregon-Henao et al., 2015; van Ingen et al., 2013).

Finding demonstrate that C3HeB/FeJ mice infected with MAC (∼2,000 organisms per mouse) – as compared to an inoculum dose of 1 × 10^8^–1 × 10^9^ organisms per mouse typically utilized with infection of mouse models (Torrelles et al., 2002; van Ingen et al., 2013; Andrejak et al., 2015; Cha et al., 2015)- resulted in a progressive infection, distinct temporal immune responses, and histopathologic changes resulting in small foci of necrosis in the lungs.

More specifically, after 20 days of infection, the C3HeB/FeJ mice showed a burden of 3.5 log_10_ CFU in the lungs followed by an increase of 1.8 log_10_ CFU during the chronic phase of disease. C3HeB/FeJ mice showed a delayed dissemination to the spleen, and liver, over the first 30 days of infection, followed by an increase of ∼4.0 log_10_ CFU in the spleen and ∼3.0 log_10_ CFU in the liver during the chronic phase of disease. An advantage of the C3HeB/FeJ model over the currently utilized MAC mouse models that use 1 × 10^8^–1 × 10^9^ CFU (Torrelles et al., 2002; van Ingen et al., 2013; Andrejak et al., 2015; Cha et al., 2015) to infect mice is that much fewer bacteria were used to infect the mice, mimicking the relative paucibacillary load seen in non-HIV infected subjects infected with NTM (Farhi et al., 1986; Hibiya et al., 2013; Kobashi et al., 2013).

Our prior studies with *M. tuberculosis* infection of C3HeB/FeJ mice demonstrated necrotic granulomas in the lungs during the chronic phase of the disease, allowing for bacterial replication, while fewer tubercular granulomas were present in the spleen and liver, leading to better bacterial clearance (Henao-Tamayo et al., 2015). However, presumably due to the reduced virulence of MAC compared to *M. tuberculosis*, MAC demonstrated a slower disease progression prior to 20 days where thereafter the development of small foci of necrosis appeared in MAC*-*infected granulomas. An additional advantage of the C3HeB/FeJ model over currently utilized MAC mouse models is the presence of small foci of necrosis in the granulomas - absent in the latter mouse strains (Torrelles et al., 2002; van Ingen et al., 2013; Andrejak et al., 2015; Cha et al., 2015) - since formation of these characteristic lesions is important in the pathogenesis of human NTM lung infections (Farhi et al., 1986; Hibiya et al., 2013; Kobashi et al., 2013).

Development of small foci of necrosis is an important aspect of NTM pathogenesis. Bacteria residing in necrotic granulomas have access to an abundant source of carbon in the form of cholesterol and triglycerides (Obregon-Henao et al., 2013), and this may be the reason for the relatively high NTM bacilli growth in these lesions. Furthermore, antibiotic penetration is significantly reduced in necrotic granulomas (Farhi et al., 1986), which could help explain the protracted antibiotic therapy required to eradicate NTMs. Thus, it is important to have these aspects in our mouse models that are utilized for compound and vaccine screening.

C3HeB/FeJ mice infected with MAC showed increased numbers of CD11b^+^ macrophages and CD11c^+^ dendritic cells expressing MHC class II during acute phase of the infection, however, this waned during the chronic phase of the disease when expression of IL-27 and PD-1L increased. Studies investigating macrophage deactivation by examining the expression of a panel of IFN-γ-inducible genes and activation of Janus kinase (JAK)-STAT pathway in MAC*-*infected macrophages showed reduced expression of IFN-γ-inducible genes—MHC class II gene Eβ; MHC class II transactivator; IFN regulatory factor-1; and Mg21, a gene coding for a GTP-binding protein in MAC*-*infected macrophages (Hussain et al., 1999). These studies support our results showing a reduction of MHC class II-expressing CD11b^+^ macrophages and CD11c^+^ dendritic cells during chronic disease (Taylor-Robinson et al., 1994).

C3HeB/FeJ mice infected with MAC was associated with increased numbers of CD3^+^CD4^+^CD44^hi^ and CD3^+^CD8^+^CD44^hi^ activated T effector cells and CD3^+^CD4^+^CD44^hi^CD127^+^ and CD3^+^CD8^+^CD44^hi^CD127^+^ memory T cells between 40 and 50 days after infection, followed by diminished numbers. In addition, C3HeB/FeJ mice were infected with MAC also demonstrated increased numbers of IFN- γ and IL-17 CD4^+^ cells as well as increased Foxp3^+^CD4^+^ T regulatory cells during late chronic infection (60 days). Interestingly, MAC-infected C3HeB/FeJ mice displayed increasing numbers of CD8^+^ T cells expressing IL-17 and Foxp3 over the course of the infection period but a modest increase of CD8^+^IFN-γ^+^ T cells. CD4^+^ T cell depleted C57BL/6 mice clearly indicate a role for CD4^+^ T cell needed for control of an intranasal infection of MAC (Florido et al., 1999). In this previous study, IFN-γ^+^ depletion before and during MAC infection led to increased bacterial burden in then lung, spleen and liver, suggesting a protective role for IFN-γ against this pathogen.

In previous studies using C57BL/6 mice and C3HeB/FeJ mice, we reported that protection waned with specific alterations of the adaptive immune response, such as increased numbers of regulatory T cells (Henao-Tamayo et al., 2015), concomitant with reduced numbers of protective CD8^+^ T cells. However, our previous studies infecting C3HeB/FeJ mice with *M. tuberculosis* (Henao-Tamayo et al., 2015) showed a reduction of both IFN-γ producing CD4^+^ and CD8^+^ T cell responses with a concomitant increase in the influx of CD4^+^ and CD8^+^ Foxp3^+^ T regulatory cells. Due to the lower dose of MAC used to infect C3HeB/FeJ mice, it is plausible that MAC disease requires additional immune deficits to display progression or a higher infection dose.

A MAC-infected C3HeB/FeJ mouse model is supported by other murine studies showing early *in vivo* expression of IFN-γ during MAC*-*infection correlated with resistance to the infection (Gangadharam, 1995; Young and Bermudez, 2001; Appelberg, 2006). The use of specific neutralizing antibodies *in vivo* led to the identification of IFN-γ and TNF-α as protective cytokines acting at the effector level of resistance to MAC (Gangadharam, 1995; Young and Bermudez, 2001; Appelberg, 2006).

## Conclusion

In conclusion, MAC infections are becoming an emerging problem worldwide. To deal with the increasing number of infected NTM patients and the resultant morbidity and mortality caused by these pathogens, multiple laboratories are focused on developing new preclinical models to screen new or repurposed compounds to fight the emergence of these pathogens. Our studies support the use of a MAC infected C3HeB/FeJ mouse model for testing candidate compounds against MAC.

## Ethics Statement

This study was carried out in accordance with the recommendations of NRC Guide for the Care and Use of Laboratory Animals (National Research Council, 2010), the requirements of the Public Health Service (PHS) Grants Administration Manual, and The Animal Welfare Act as amended. CSU files assurances with the DHHS Office of Extramural Research, Office of Laboratory Animal Welfare (OLAW), the Public Health Service, and adheres to NIH standards and practices for grantees. The protocol was approved by the Colorado State Universities Animal Care and Usage Committee.

## Author Contributions

DO, EC, AS, DV, and MS conceived and designed the study, acquired, analyzed, and interpreted the data, and drafted the manuscript. JG, KV, and AT analyzed and interpreted the data, and drafted and revised the manuscript. DO, EC, AS, and DV conceived and designed the study, analyzed and interpreted the data, and drafted, revised, and approved the manuscript.

## Dedication

In memory of Dr. Ian Orme.

## Conflict of Interest Statement

The authors declare that the research was conducted in the absence of any commercial or financial relationships that could be construed as a potential conflict of interest.
